# High‐Performance Flexible Quasi‐Solid‐State Supercapacitors Realized by Molybdenum Dioxide@Nitrogen‐Doped Carbon and Copper Cobalt Sulfide Tubular Nanostructures

**DOI:** 10.1002/advs.201800733

**Published:** 2018-08-11

**Authors:** Shude Liu, Ying Yin, Kwan San Hui, Kwun Nam Hui, Su Chan Lee, Seong Chan Jun

**Affiliations:** ^1^ School of Mechanical Engineering Yonsei University Seoul 120‐749 South Korea; ^2^ Guangxi Key Laboratory of Information Materials Guilin University of Electronic Technology Guilin 541004 P. R. China; ^3^ School of Mathematics University of East Anglia Norwich NR4 7TJ UK; ^4^ Institute of Applied Physics and Materials Engineering University of Macau Avenida da Universidade Taipa Macau 999078 China

**Keywords:** CuCo_2_S_4_, electrochemical performance, flexible quasi‐solid‐state supercapacitors, MoO_2_, tubular nanostructures

## Abstract

Flexible quasi‐/all‐solid‐state supercapacitors have elicited scientific attention to fulfill the explosive demand for portable and wearable electronic devices. However, the use of electrode materials faces several challenges, such as intrinsically slow kinetics and volume change upon cycling, which impede the energy output and electrochemical stability. This study presents well‐aligned molybdenum dioxide@nitrogen‐doped carbon (MoO_2_@NC) and copper cobalt sulfide (CuCo_2_S_4_) tubular nanostructures grown on flexible carbon fiber for use as electrode materials in supercapacitors. Benefiting from the chemically stable interfaces, affluent active sites, and efficient 1D electron transport, the MoO_2_@NC and CuCo_2_S_4_ nanostructures integrated on conductive substrates deliver excellent electrochemical performance. A flexible quasi‐solid‐state asymmetric supercapacitor composed of MoO_2_@NC as the negative electrode and CuCo_2_S_4_ as the positive electrode achieves an ultrahigh energy density of 65.1 W h kg^−1^ at a power density of 800 W kg^−1^ and retains a favorable energy density of 27.6 W h kg^−1^ at an ultrahigh power density of 12.8 kW kg^−1^. Moreover, it demonstrates good cycling performance with 90.6% capacitance retention after 5000 cycles and excellent mechanical flexibility by enabling 92.2% capacitance retention after 2000 bending cycles. This study provides an effective strategy to develop electrode materials with superior electrochemical performance for flexible supercapacitors.

## 1 Introduction

Supercapacitors are an attractive choice for electrochemical energy storage due to their longer cycling lifetimes and higher power densities than rechargeable lithium ion batteries.^1^, ^2^ With the continuous increase in the consumption of portable and wearable electronic devices, flexible solid‐state supercapacitors have been eliciting considerable attention recently because of their notable features, such as lightweight, environment friendliness, and good mechanical properties.^3^ However, the energy densities of most commercially available supercapacitors are much lower than those of rechargeable batteries, and this feature poses a significant obstacle in the large‐scale practical application of such supercapacitors. Energy density is related to specific capacitance *C* and operating voltage window *V*, i.e., *E* = $\frac{1}{2}C\cdot V^{2}$.^1^, ^4^ Hence, extensive effort has been exerted to maximize *E* by increasing *V* and/or *C*.^4^ Asymmetric supercapacitors (ASCs) offer an effective strategy to extend cell voltage contributions from separate potential windows of negative and positive electrode materials, thereby providing favorable power sources to meet the energy demands of emerging electronic devices.^1^, ^5^, ^6^ Thus, the search for highly capacitive electrode materials has inspired extensive research attention.

The energy densities of current ASC devices are constrained by negative electrode materials with low specific capacitances.^7^ Therefore, alternative materials with high specific capacitances are desired. Molybdenum dioxide (MoO_2_) presents considerable promise as a negative electrode material for supercapacitors because of its high theoretical specific capacitance, multiple molybdenum valence states, and environmental benignity.^8^, ^9^ Nevertheless, many experimentally obtained MoO_2_ nanostructures are limited by intrinsically sluggish electrochemical kinetics and large volume expansion and contraction upon cycling, leading to inferior rate capability and rapid performance degradation upon cycling.^2^ The use of hollow structures for energy storage has attracted much attention owing to the attractive structure features of high surface‐to‐volume ratio, short‐charge transport lengths, abundant electrochemical active sites accessible to the electrolyte, and well‐defined interior space.^10^ In particular, hierarchical tubular nanostructures synergistically combine the advantages of low‐dimensional building blocks and hollow structures, thus exhibiting enhanced electrochemical performance.^11^ However, hollow metal oxide structures inevitably suffer from intrinsically low electrical conductivity, which leads to pulverization during cycling tests. To resolve this challenge, researchers have combined these structures with carbonaceous materials to create a feasible strategy for ameliorating the electrochemical performance of transition metal materials.^12^ The incorporation of N heteroatoms in carbon has been proven effective to modulate the bandgap and optimize surface characteristics, allowing fast electron transfer and electrolyte ion diffusion kinetics.^13^, ^14^ Moreover, the existence of N contents in carbon enhances the hydrophilicity for electrolyte transportation in comparison with bare carbon materials. From the perspective of electrode design, the fabrication of 3D electrodes with mechanically/chemically stable interfaces not only ensures favorable ion/electron transport pathways but also avoids the use of additional binders and conductive agents.^15^ The integral design of hierarchical MoO_2_ tubular nanostructure/N‐doped carbon hybrids on conductive lightweight carbon substrates is highly desirable for flexible supercapacitors but has been rarely reported.

The specific capacitance, *C*_T_, of asymmetric devices is determined from positive (*C*_+_) and negative (*C*_−_) electrode material specific capacitances, i.e., 1/*C*_T_ = 1/*C*_+_ + 1/*C*_−_. Various strategies have been proposed to develop high‐capacitive positive electrode materials.^16^ Transition metal sulfides are promising supercapacitor candidates because M—S bonds in metal sulfides are weaker than M—O bonds in metal oxides and are thus more kinetically reversible.^17^ In particular, mixed metal sulfides with introduced heterometal ions provide richer redox reactions and better electrical conductivity than single‐component sulfides, which can be favorable for enhanced electrochemical performance.^18^

Stimulated by these considerations, we successfully synthesized molybdenum dioxide@nitrogen‐doped carbon (MoO_2_@NC) and copper cobalt sulfide (CuCo_2_S_4_) tubular nanostructures on conductive carbonaceous backbones as superior electrode materials for supercapacitors. Flexible quasi‐solid‐state asymmetric supercapacitors were fabricated using MoO_2_@NC and CuCo_2_S_4_ as positive and negative electrodes, respectively. The assembled device exhibited a maximum energy density of 65.1 W h kg^−1^ at a specific power of 800 W kg^−1^ and retained a maximum power density of 12.8 kW kg^−1^ at a specific energy of 27.6 W h kg^−1^. Approximately 90.6% of the initial specific capacitance could be retained after 5000 cycles, and the device could be bent for 2000 times with a capacitance retention of 92.2%. This study reveals the potential use of integrated tubular architectures for high‐performance flexible electrochemical energy storage applications.

## 2 Results and Discussion

### 2.1 Characterization and Electrochemical Properties of the Negative Electrode Material

**Figure 1:**
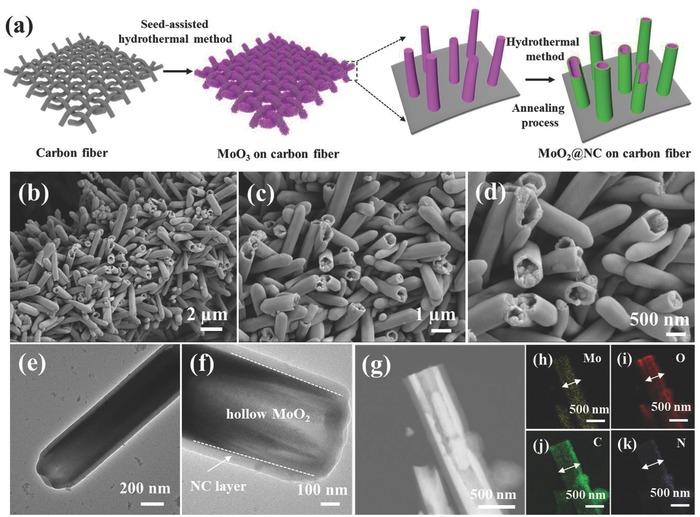
a) Schematic of the process of MoO_2_@NC synthesis on carbon fiber. b–d) SEM images of MoO_2_@NC, e,f) TEM images of MoO_2_@NC, g) STEM image, and h–k) corresponding EDS mappings of MoO_2_@NC.

Hierarchical MoO_2_@NC tubular nanostructure was synthesized on flexible carbon fiber through a two‐step process (**Figure** **1**a). First, MoO_3_ nanorods were deposited on carbon fiber via a seed‐assisted hydrothermal method. Scanning electron microscopy (SEM) images (Figure S1a–c, Supporting Information) showed that the entire substrate surface was uniformly covered by MoO_3_ nanorods with 400–600 nm diameter. Energy dispersive X‐ray spectrometry (EDS) showed the existence of Mo and O elements (Figure S1d–g, Supporting Information). Second, MoO_2_@NC was fabricated through one‐step hydrothermal reaction in glucosamine hydrochloride solution followed by annealing,^19^, ^20^ wherein MoO_3_ was reduced to MoO_2_ and N‐containing carbon coating was formed. The results showed that the outer surfaces of the hollow MoO_2_ structure were decorated with an N‐containing carbon layer, and its structural orientation was well preserved (Figure 1b–d). The low‐magnification SEM image and corresponding EDS mappings showed uniform Mo, O, C, and N elemental distribution throughout the MoO_2_@NC structure (Figure S2, Supporting Information). Low‐magnification transmission electron microscopy (TEM) (Figure 1e–f) showed the tubular structure by distinct contrast between core and shell regions. Scanning TEM (STEM) and corresponding EDS mappings (Figure 1g–k) showed that Mo and O signals were mainly distributed in the core region, whereas N and C signals were primarily found in the shell region. The widened N and C signal distributions confirmed that N‐containing carbon was closely attached on the MoO_2_ surface, thus confirming the MoO_2_@NC hierarchical tubular nanostructure.

**Figure 2:**
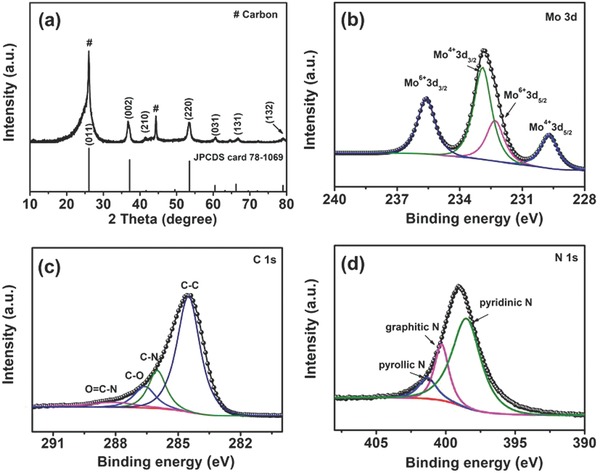
a) XRD pattern of MoO_2_@NC. High‐resolution XPS spectra of b) Mo 3d, c) C 1 s, and d) N 1 s for MoO_2_@NC.

The crystal phases of the as‐synthesized samples were investigated by X‐ray diffraction (XRD). All of the diffraction peaks of MoO_3_ nanorods were readily assigned to the hexagonal MoO_3_ phase (JCPDS no. 21‐569) aside from the two peaks at 2θ = 26.2° and 44.4° corresponding to the carbon substrate (Figure S3, Supporting Information).^21^, ^22^ **Figure** **2**a shows that MoO_2_@NC diffraction peaks exhibited good agreement with monoclinic MoO_2_ phase (JCPDS no. 73‐1069),^23^ and the other peaks could be indexed to the carbon structure.^22^ The crystal structure of MoO_2_@NC placed in air for around six months was examined by XRD analysis (Figure S4, Supporting Information). It can be observed that no change in crystal structure was detected compared to that of MoO_2_@NC placed in air for two weeks, implying good thermal stability of MoO_2_@NC. Raman spectroscopy was applied to analyze the phase transition of the as‐prepared samples. The Raman spectrum of MoO_3_ (Figure S5a, Supporting Information) is highly consistent with the structural features of previously reported MoO_3_.^24^ After hydrothermal carbonization and reduction, MoO_2_@NC exhibited MoO_2_ plane reflections with two characteristic peaks corresponding to the D band (1353 cm^−1^) and G band (1595 cm^−1^) of carbon (Figure S5b, Supporting Information).^25^, ^26^, ^27^ The surface chemical states of MoO_3_ and MoO_2_@NC were further studied by X‐ray photoelectron spectroscopy (XPS). The Mo 3d core‐level spectrum of MoO_3_ (Figure S6, Supporting Information) had a spin–orbit doublet with peaks at 232.8 eV for Mo 3d_5/2_ and 236.0 eV for Mo 3d_3/2_ together with an energy separation of 3.2 eV, which is consistent with previous reports on Mo^6+^ in MoO_3_.^28^ For MoO_2_@NC, the Mo 3d peak was deconvoluted into four peaks (Figure 2b). The two peaks centered at 229.7 and 232.9 eV were indexed to Mo 3d_5/2_ and Mo 3d_3/2_ of Mo^4+^, respectively.^9^ The two other peaks located at 232.3 and 235.8 eV corresponded to Mo 3d_5/2_ and Mo 3d_3/2_ of Mo^6+^, which could be ascribed to MoO_2_ surface oxidation under ambient conditions.^29^, ^30^ The collected C 1s spectrum of MoO_2_@NC (Figure 2c) showed bands at 284.6, 285.7, 286.4, and 287.7 eV corresponding to C—C, C—N, C—O, and O=C—N bonds, respectively.^31^, ^32^ These types of surface functional groups in the carbon shell may contribute to enhanced electrochemical reaction kinetics.^33^ For the high‐resolution N 1s spectrum of MoO_2_@NC (Figure 2d), three deconvoluted peaks were observed at binding energies of 398.5, 400.3, and 401.3 eV corresponding to pyridinic N, pyrrolic N, and graphitic N, respectively,^34^, ^35^ indicating the successful incorporation of nitrogen into graphitic carbon.

**Figure 3:**
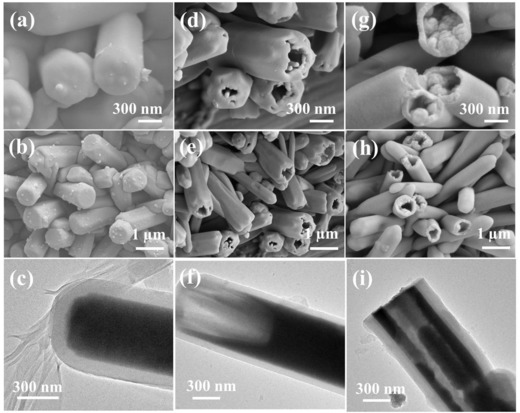
Typical FESEM and TEM images of MoO_2_@NC obtained after hydrothermal reaction for a–c) 12 h, d–f) 18 h, and g–i) 24 h.

The time‐dependent morphology evolution process was conducted to investigate the formation of the hierarchical MoO_2_@NC tubular structure (**Figure** **3**). In the initial process (12 h), the formed carbon layers were homogeneously deposited on the exterior surface of MoO_2_ through facile hydrothermal carbonization and reduction (Figure 3a–c). The 1D morphology was well preserved as the reaction proceeded (18 h), with narrow voids existing between internal MoO_2_ core and external carbon shell (Figure 3d–f). The core was gradually eroded (24 h) and finally produced an almost completely hollow MoO_2_@NC nanostructure (Figure 3g–i). The formation mechanism of hollow‐structured MoO_2_@NC was as follows. Glucosamine hydrochloride not only served as an N‐containing carbon precursor but also provided a weak acidic medium.^36^ During the hydrothermal reaction, a thin protective layer of carbon was formed by hydrothermally carbonized glucosamine on the surface of MoO_3_, while MoO_3_ was concurrently reduced into MoO_2_ due to reducibility of glucosamine during hydrothermal process.^19^ MoO_3_ is stable in weak acidic environments,^37^ whereas MoO_2_ is dissolved in acidic conditions.^38^, ^39^ The as‐synthesized MoO_2_ nanorods were gradually eroded from the interior instead of the exterior because the carbonized carbon layer could efficiently protect the exterior of the MoO_2_ surface.

**Figure 4:**
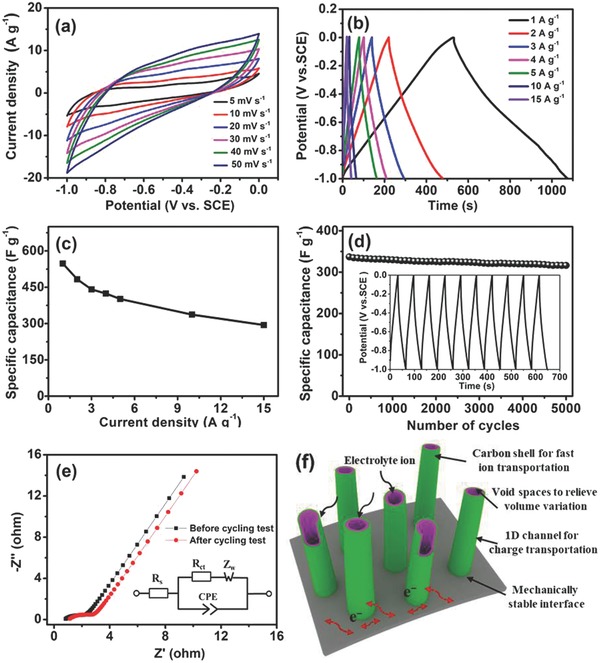
Electrochemical characterization of MoO_2_@NC as the electrode material for supercapacitors: a) CV profiles at different scan rates; b) GCD profiles at different current densities; c) specific capacitance versus current density; d) cycling performance measured at a current density of 10 A g^−1^, the inset is the last 10 cycles during the cycling test; e) Nyquist plots before and after the cycling test, the inset is the proposed equivalent circuit; f) schematic of the structural advantages of MoO_2_@NC on carbon cloth for electrochemical energy storage properties.

The electrochemical property of MoO_2_@NC as the negative electrode was evaluated using a three‐electrode configuration in 1 m KOH aqueous electrolyte. The cyclic voltammetry (CV) curves of MoO_2_@NC electrode at various scan rates were examined (**Figure** **4**a). The shape of the CV curves deviated from the ideal rectangular CV curves primarily due to the Faradaic redox reaction of Mo^4+^/Mo^6+^ and reversible intercalation/de‐intercalation of K^+^ ions from the host.^40^, ^41^, ^42^, ^43^ No obvious shape deformation was detected in these CV curves of MoO_2_@NC, with the scan rate extended from 5 to 50 mV^−1^, implying a high rate capability and low internal resistance. Notably, the enclosed area of the CV curve for carbon fiber was negligible when compared with that for the MoO_2_@NC electrode, indicating that the capacitance contribution of the substrate can be ignored (Figure S7, Supporting Information).

Figure 4b shows galvanostatic charge–discharge (GCD) curves for the as‐fabricated MoO_2_@NC at different current densities. The symmetric triangular shapes of the GCD curves manifested the high reversibility and capacitive behavior of MoO_2_@NC,^44^ consistent with the CV curves. Specific capacitance as a function of current density was calculated from the GCD curves (Figure 4c). The MoO_2_@NC electrode displayed a specific capacitance of 548 F g^−1^ at a current density of 1 A g^−1^. To the best of our knowledge, this is the largest reported specific capacitance for MoO_2_ nanostructures and MoO_2_/carbon hybrids (Table S1, Supporting Information). The MoO_2_@NC electrode can still reserve a specific capacitance of 294 F g^−1^ even when the current density increased to 15 A g^−1^. The cycling performance of the MoO_2_@NC electrode was investigated at a current density of 10 A g^−1^ (Figure 4d). Remarkably, the MoO_2_@NC electrode exhibited excellent electrochemical stability with 93.9% capacitance retention after 5000 cycles. Electrochemical stability was confirmed by examining stable GCD curves for the last 10 cycles (inset of Figure 4d). Electrochemical impedance spectroscopy (EIS) was conducted to provide further insights into the excellent electrochemical performance of MoO_2_@NC (Figure 4e). The Nyquist plot of the MoO_2_@NC electrode was analyzed with the proposed equivalent circuit (inset of Figure 4e). The small bulk resistance (*R*_s_) of the electrochemical system was calculated to be 0.83 Ω cm^−2^ in the high‐frequency region. The representative semicircle corresponding to charge‐transfer resistance (*R*_ct_) was 1.96 Ω cm^−2^, suggesting good ionic conductivity at the electrode/electrolyte interface. The curve shapes of EIS spectra before and after the cycling tests were similar, except for a small increase in the equivalent series resistance. The excellent electrochemical properties of MoO_2_@NC can be attributed to the following merits (Figure 4f). 1) This integrated configuration involving the direct growth of MoO_2_@NC on highly conductive textile not only avoids the increase in electrode resistance caused by the addition of polymer binders and carbon additives but also allows for efficient electrolyte accessibility and electron movement, thus ensuring the utilization of the active materials. 2) The longitudinal axis of 1D nanostructures guarantees efficient transport of electrons and ions. 3) The well‐defined inner voids embedded in the highly conductive carbon matrix not only function as an “electrolyte container” to create sufficient electron paths for rapid transportation of electrolyte ions but also alleviate volume shrinkage/expansion during the charge/discharge process to prevent structural collapse. 4) The introduction of N‐doped carbon shell accommodates the volumetric change, improves the electrical conductivity of carbon, and facilitates effective transport of electrons, thus preventing a rapid decrease in capacity.

### 2.2 Characterization and Electrochemical Properties of the Positive Electrode Material

**Figure 5:**
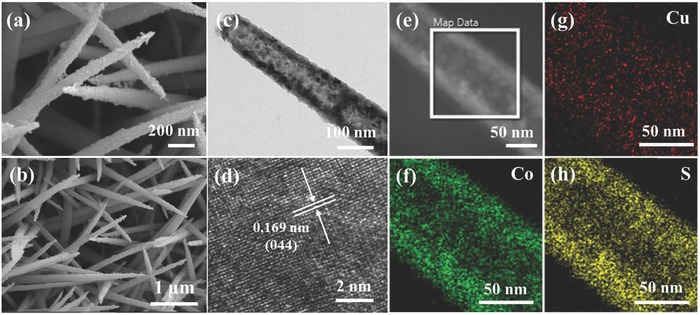
Electron microscopy characterization of the CuCo_2_S_4_ tubular nanostructure: a,b) SEM images; c) TEM image; d) HRTEM image; e) STEM image, and f–h) corresponding EDS mappings of the designated area in the STEM image.

A representative SEM image (Figure S8a, Supporting Information) showed that Cu–Co precursor nanowires were grown uniformly on the entire surface of carbon cloth. Close observation (Figure S8b,c, Supporting Information) showed that the nanowires with smooth surfaces had a length of ≈3 µm and a diameter of ≈100 nm. The wire morphology was retained after sulfurization, and the surface of the nanostructure became rough and presented a hollow structure (**Figure** **5**a,b and Figure S8d (Supporting Information)). The structural change can be attributed to the etching effect of sulfur ions and the nonequilibrium diffusion rate between the outward cationic species and inward sulfur ions.^45^ A representative SEM image of CuCo_2_S_4_ and the corresponding EDS elemental mappings (Figure S8e–I, Supporting Information) confirmed the coexistence of Cu, Co, and S elements in the sample. The TEM image (Figure 5c) clearly showed the tubular structure by well‐defined inner voids between the center and edge. The spacing of lattice fringes in the high‐resolution TEM (HRTEM) image was 0.169 nm (Figure 5d), corresponding to the (044) plane of the CuCo_2_S_4_ crystal. The STEM image of CuCo_2_S_4_ (Figure 5e) and corresponding element mappings (Figure 5g,h) from the designated area confirmed the tubular nanostructure.

**Figure 6:**
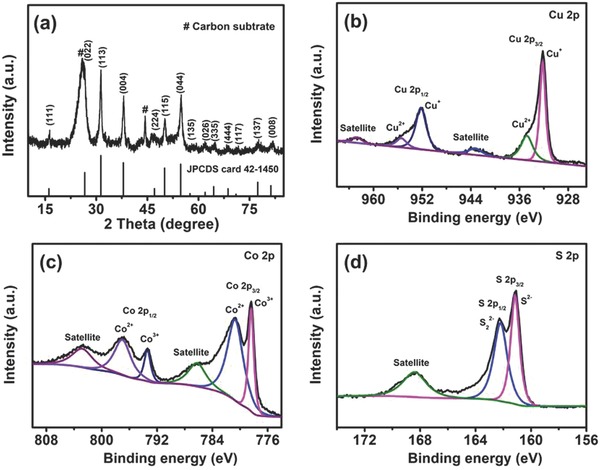
a) XRD pattern of the CuCo_2_S_4_ nanostructure. High‐resolution XPS spectra of b) Cu 2p, c) Co 2p, and d) S 2p for the CuCo_2_S_4_ nanostructure.

The crystal phase of the product was investigated by the XRD technique (**Figure** **6**a). All diffraction peaks in the XRD spectrum were well indexed to cubic CuCo_2_S_4_ (JCPDS card No. 42‐1450)^46^ and the carbon substrate (JCPDS card No. 75‐1621).^22^ Furthermore, XPS was performed to analyze the surface chemical compositions and valence states of CuCo_2_S_4_. The full survey spectrum revealed the existence of Cu, Co, and S elements (Figure S9, Supporting Information), consistent with the EDS analysis. The detected atomic ratio of Cu, Co, and S was ≈1:1.97:3.76 in the sample, further confirming the component of CuCo_2_S_4_ species. In the high‐resolution Cu 2p spectrum (Figure 6b), the two peaks at 932.2 and 952.2 eV corresponded to the characteristic of Cu^+^. The two other peaks at 934.8 and 955.4 eV with two shake‐up satellites located at 943.3 and 962.8 eV, respectively, were assigned to the signal of Cu^2+^,^46^, ^47^ which originates from air exposure.^48^ The Co 2p spectrum (Figure 6c) was best fitted with two spin–orbit doublets and several shake‐up satellite characteristics of Co^3+^ and Co^2+^ species. The fitted peaks at binding energies of 778.4 and 793.4 eV with a spin–orbit splitting of 15 eV indicated the presence of Co^3+^,^48^, ^49^ whereas the peaks at 780.7 and 797.0 eV were assigned to Co^2+^. The S 2p spectrum (Figure 6d) can be deconvoluted into two major peaks of S 2p_3/2_ and S 2p_1/2_ and one shake‐up satellite peak. The peaks centered at 161.1 and 162.2 eV can be attributed to S^2−^ and the sulfion in low coordination (S_2_^2−^) at the surface, respectively.^49^, ^50^, ^51^

**Figure 7:**
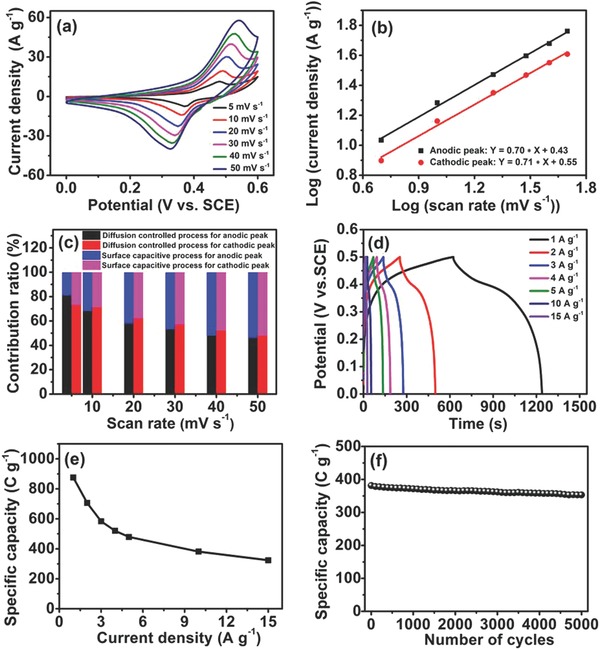
Electrochemical performance of CuCo_2_S_4_ as a positive electrode in a three‐electrode configuration: a) CV curves at different scan rates from 5 to 50 mV s^−1^; b) plots of log (scan rate) versus log (peak current) calculated from CV curves; c) contribution ratios of diffusion‐controlled and surface capacitive charge at different scan rates for anodic and cathodic peaks; d) GCD curves at different current densities; e) specific capacity as a function of current density; f) cycling performance conducted at a current density of 10 A g^−1^.

The electrochemical performance of CuCo_2_S_4_ was investigated using a three‐electrode system in 1 m KOH electrolyte. The CV profiles at different scan rates showed a couple of well‐defined characteristic redox peaks (**Figure** **7**a) corresponding to the Faradaic reactions of Cu^2+^/Cu^+^ and Co^4+^/Co^3+^/Co^2+^ related to OH^−^ in the electrolyte. The involved redox reactions can be illustrated by the following equations^46^, ^52^, ^53^, ^54^

(1) $$\mathrm{CuS}\;+\;{\mathrm{OH}}^{-}\;↔\;\mathrm{CuSOH}\;+\;e^{-}$$

(2) $$\mathrm{CoS}\;+\;{\mathrm{OH}}^{-}\;↔\;\mathrm{CoSOH}\;+\;e^{-}$$

(3) $$\mathrm{CoSOH}\;+\;{\mathrm{OH}}^{-}\;↔\;\mathrm{CoSO}\;+\;e^{-}\;+\;H_{2}O$$

A small shift was observed in the redox peaks when the scan rate increased from 5 to 50 mV s^−1^, implying rapid reaction kinetics. To investigate the charge storage mechanism of CuCo_2_S_4_, we analyzed the relationship between measured current (*i*) and scan rate (*v*) by using the power law (*i*  =  *av^b^*).^55^ The value of *b* was determined by the slope of log(*v*)–log(*i*) plot. The *b*‐value of 0.5 represents a diffusion‐controlled behavior, and a value of 1.0 indicates a capacitive process.^56^ The obtained *b*‐values were 0.70 and 0.71 for anodic and cathodic peaks, respectively (Figure 7b), suggesting a dominant diffusion‐controlled process for charge storage.^57^ The capacitive contribution of capacitor‐like and diffusion‐controlled behaviors at a certain scan rate (*v*) can be quantified according to the formula *i*_c_ = *k*_1_ *v*  +  *k*_2_ *v* ^0.5^,^58^ where *i*_c_, *k*_1_, and *k*_2_ are the current response of the redox peak and the fractions of current contributed by capacitive and diffusion‐controlled processes, respectively. The quantified results (Figure 7c) showed that the surface capacitive contributions of the anodic and cathodic peaks increased with the increase in scan rate and finally reached the maximum values of 54% and 52% at 50 mV s^−1^, respectively.

Figure 7d shows nearly symmetric GCD curves at different current densities, suggesting a highly reversible behavior of CuCo_2_S_4_. It can be seen that GCD curves exhibited obvious voltage plateaus, indicating the typical Faradaic behavior of battery‐type materials.^59^ The calculated specific capacity was 875 C g^−1^ at a current density of 1 A g^−1^ (Figure 7e), which outperforms those of previously developed CuCo_2_S_4_ nanostructures at the same current density.^46^, ^60^ Moreover, CuCo_2_S_4_ could retain 323 C g^−1^ when the current density increased to 15 A g^−1^. The EIS result (Figure S10a, Supporting Information) showed a low *R*_s_ (0.91 Ω cm^−2^) and *R*_ct_ (1.72 Ω cm^−2^), implying high electrical conductivity and rapid electron transport kinetics. After the cycling test, *R*_ct_ showed a small increase (Figure S10b, Supporting Information), illustrating that the CuCo_2_S_4_ nanostructure was well preserved, which is consistent with the stable cycling performance. Cycling stability was evaluated by conducting consecutive GCD tests at a current density of 10 A g^−1^ (Figure 7f). A capacity retention of 92.8% was achieved after 5000 cycles, which is much higher than those in several latest reports on ternary transition metal sulfides.^46^, ^61^, ^62^ It is clear that the structural and crystalline phase of CuCo_2_S_4_ was preserved after cycling test except the newly formed peaks at around 19.1° and 51.7° corresponding to the presence of Co(OH)_2_ (Figure S11, Supporting Information), which is ascribed to surface electrochemical oxidation during the long‐term cycling process consistent with previous report.^63^

### 2.3 Electrochemical Performance of the Flexible Quasi‐Solid‐State ASC Device

**Figure 8:**
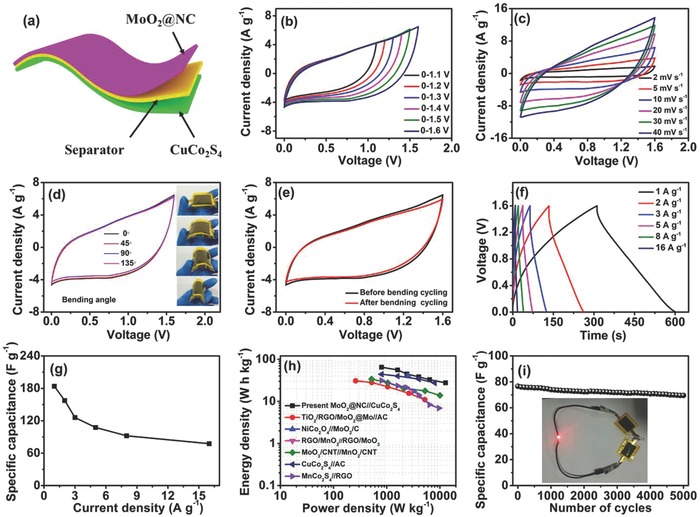
Electrochemical characterization and flexibility analysis of the as‐assembled MoO_2_@NC//CuCo_2_S_4_ ASC device: a) schematic of the device configuration; b) CV curves obtained in different potential windows at 10 mV s^−1^; c) CV curves at different scan rates; d) comparative CV curves at different curvatures, the inset is the optical image of the assembled ASC device at different bending states; e) comparative CV curves before and after bending the substrate at 90° for 2000 times; f) GCD curves collected at different current densities; g) specific capacitance at different current densities; h) Ragone plot related to energy and power densities; i) cyclic stability over 5000 cycles at 16 A g^−1^ (the inset shows the digital image of LED lighted by two ASC devices connected in series).

A flexible quasi‐solid‐state ASC device with polyvinyl alcohol (PVA)–KOH gel electrolyte was assembled using the as‐prepared MoO_2_@NC as the negative electrode and CuCo_2_S_4_ as the positive electrode (**Figure** **8**a). To achieve the optimal electrochemical performance of the device, the mass ratio of MoO_2_@NC to CuCo_2_S_4_ was set to ≈0.5 according to the CV curves of the two electrodes at a scan rate of 10 mV s^−1^ (Figure S12, Supporting Information) using charge balance theory.^64^ Figure 8b shows the CV curves of the device recorded at a scan rate of 10 mV s^−1^ at different voltage windows. As expected, no obvious irreversible current was observed in the CV curves until the cell voltage increased to 1.6 V, indicating that a stable cell voltage window could be extended to 1.6 V. The shape of the CV curves realized in the wide voltage window could be ascribed to the synergetic contributions of the two electrodes. Figure 8c shows the typical CV curves recorded at various scan rates ranging from 2 to 40 mV s^−1^. No obvious deformation was noted in the CV curves, indicating a good rate capability.^65^ To illustrate the flexibility of the device, electrochemical performance was characterized by acquiring the CV curve at different bending angles (Figure 8d). The shape of the CV curves recorded at a scan rate of 10 mV s^−1^ was nearly invariable under different degrees of bending (0°, 45°, 90°, and 135°). After bending at 90° for 2000 cycles, the device still retained 92.2% of its original capacitance (Figure 8e). These results indicate the great potential of using the present MoO_2_@NC//CuCo_2_S_4_ as an alternative wearable electronic device.

The GCD curves of the device showed that the potential–time curves were nearly symmetric at different current densities (Figure 8f), indicating high Coulombic efficiency.^66^ The calculated specific capacitance as a function of current density is presented in Figure 8g. The device delivered a maximum specific capacitance of 184 F g^−1^ at a current density of 1 A g^−1^, while 78 F g^−1^ was retained when the current density increased to 16 A g^−1^. Figure 8h shows the Ragone plot of the device in relation to energy and power densities. The maximum gravimetric energy density reached 65.1 W h kg^−1^ at a power density of 800 W kg^−1^, and a favorable energy density of 27.6 W h kg^−1^ was retained even at a high power density of 12.8 kW kg^−1^. These values are superior to the values of previously reported devices in literature, such as TiO_2_/reduced graphene oxide (RGO)/MoO_2_@Mo//activated carbon (AC),^67^ NiCo_2_O_4_//MoO_2_/C,^68^ RGO/MnO_2_//RGO/MoO_3_,^69^ MoO_3_/carbon nanotube (CNT)//MnO_2_/CNT,^70^ CuCo_2_S_4_//AC,^71^ and MnCo_2_S_4_//RGO.^62^

The EIS analytical result of the MoO_2_@NC//CuCo_2_S_4_ device showed that the Nyquist plot consisted of a quasi‐semicircle in the high‐frequency region followed by a straight line in the low‐frequency region (Figure S13, Supporting Information). This result reveals the combination of rapid electron transmission and low electrotransfer resistance of the device. The cycling performance of the device was investigated at a high discharge current density of 16 A g^−1^ for 5000 repetitive cycles (Figure 8i). The corresponding Coulombic efficiency exceeded 90.6% during the entire cycling processes (Figure S14, Supporting Information). The small drop of Coulombic efficiency for the device is ascribed to the increased internal resistance, which leads to the nonreversible reactions within the device.^72^, ^73^ The device retained 90.6% of its initial specific capacitance after the cycling test, illustrating good cycling stability. This cycling performance is significantly better than that of flexible devices reported in literature.^74^, ^75^, ^76^ The inset of Figure 8i shows that two assembled devices in series could illuminate light‐emitting diode (LED) of ≈2.0 V, which illustrates a practical application.

## 3 Conclusions

We developed hierarchical MoO_2_@NC and CuCo_2_S_4_ tubular nanostructures on conductive carbon fabric. The integrated electrode architectures of MoO_2_@NC and CuCo_2_S_4_ on a flexible carbon substrate exhibited good mechanical robustness, short ion diffusion paths, large active surface area, and good capability to regulate volume change during cycling, resulting in excellent electrochemical performance. The flexible solid‐state asymmetric supercapacitor composed of MoO_2_@NC and CuCo_2_S_4_ as positive and negative electrodes, respectively, achieved an ultrahigh energy density of 65.1 W h kg^−1^ at a power density of 800 W kg^−1^, good electrochemical cycling stability (90.6% retention after 5000 cycles), and superior mechanical flexibility with 92.2% retention of the initial capacitance after 2000 bending cycles. These results provide valuable insights into the potential of using tubular nanostructures for high‐performance flexible energy storage devices.

## 4 Experimental Section

*Material Synthesis—Synthesis of MoO_3_ Nanorods*: Prior to the experiment, the carbon fiber was ultrasonically cleaned in acetone, ethanol, and distilled water for 10 min each. The MoO_3_ nanorods were synthesized through a reported seed‐assisted hydrothermal process with some modifications.^77^ First, NaMoO_4_·2H_2_O (4 g) was dispersed in a mixed solution containing concentrated HCl (10 mL, 37 wt%) and deionized water (40 mL). The pretreated carbon fiber was immersed in the above solution for 10 min, and taken out, dried by compressed air blowing, and in an oven at 300 °C for 5 min under ambient atmosphere. The MoO_3_ nanoparticles were successfully deposited on the carbon fiber. Second, (NH_4_)_6_Mo_7_O_24_ (1 g) was dissolved in a mixture consisting of HNO_3_ (6 mL) and distilled water (34 mL). The obtained precursor solution and the carbon fiber with deposited MoO_3_ nanoparticles were transferred to a 100 mL Teflon‐lined stainless steel autoclave and maintained at 150 °C for 10 min, followed by natural cooling. The resulting carbon fiber with MoO_3_ nanorods was washed with distilled water and ethanol several times and then dried at 80 °C for 12 h. The mass loading of MoO_3_ was about 1.1 mg cm^−2^.

*Material Synthesis—Synthesis of MoO_2_@NC Tubular Nanostructure*: The synthesized MoO_3_ nanorods on carbon fiber were placed in a homogeneous solution containing glucosamine hydrochloride (0.6 g) and deionized water (50 mL), which were transferred to a 100 mL Teflon‐lined stainless‐steel autoclave. The autoclave was sealed and maintained at 180 °C for different hydrothermal reaction times (12, 18, and 24 h) and cooled to ambient temperature naturally. The carbon fiber with MoO_2_@NC was taken out and washed with ethanol several times, followed by heat treatment at 600 °C for 4 h under Ar atmosphere.

*Material Synthesis—Synthesis of CuCo_2_S_4_ Tubular Nanostructure*: In a typical process, 2 × 10^−3^ m of Cu(NO_3_)_2_·3H_2_O, 4 × 10^−3^ m of Co(NO_3_)_2_·6H_2_O, 12 × 10^−3^ m of CO(NH_2_)_2_, and 12 × 10^−3^ m of NH_4_F were dissolved in 70 mL of deionized water and stirred to form a homogeneous pink solution. The obtained mixture and cleaned carbon cloth were transferred to a Teflon‐lined stainless‐steel autoclave and kept at 140 °C for 6 h. The obtained Cu–Co precursor on the carbon cloth was rinsed and dried at 70 °C for 12 h. Subsequently, the carbon cloth–supported Cu–Co precursor was immersed into 0.02 m of Na_2_S aqueous solution and then transferred to an autoclave and maintained at 160 °C for 6 h. The resulting product was washed with deionized water and ethanol several times and dried at 70 °C for 12 h.

*Material Characterization*: Crystal structures were characterized by XRD (Bruker D8 Advance, Cu Kα, λ = 1.5406 Å). XPS (VG Scientifics ESCALAB250) spectra were used to examine surface elemental composition and valence state. The morphology and structural properties were characterized using a field emission scanning electron microscope (FESEM, JEOL, JSM‐7800) and a transmission electron microscopy (TEM, JEOL, JSM‐2100F). Raman spectra were measured using LabRAM Aramis equipment from Horriba Jovin Yvon (laser wavelength of 532 nm).

*Electrochemical Measurements*: The electrochemical properties of the samples were measured with an IVIUM electrochemical workstation system (the Netherlands) in a three‐electrode electrochemical cell with 1 m KOH as the electrolyte, in which a platinum plate served as the counter electrode and saturated calomel as the reference electrode. The mass loadings of MoO_2_@NC and CuCo_2_S_4_ were about ≈1.4 and 0.9 mg cm^−2^, respectively. The as‐prepared samples directly acted as the working electrode. EIS measurements were carried out under open‐circuit voltage in the frequency range of 100 kHz–0.01 Hz. The specific capacitance (*C*_s_, F g^−1^) of MoO_2_@NC and specific capacity (*C*, C g^−1^) of CuCo_2_S_4_ were calculated from the GCD curves according to the following equations^59^, ^78^

(4) $$C_{s}\;=\;\frac{I\cdot \Delta t}{m\;\cdot \;\Delta V}$$

and

(5) $$C\;=\;\frac{I\int_{0}^{\Delta t}Vdt}{m\;\times \;\Delta V_{\mathrm{mean}}\;}\;=\;\frac{I\int_{0}^{\Delta t}Vdt}{m\;\times \;\frac{\Delta V}{2}\;}\;=\;2\frac{I\int_{0}^{\Delta t}Vdt}{m\Delta V\;}$$

where *I* (A) is the discharge current, Δ*V* (V) is the potential window, *m* (g) is the mass loading, *V* (V) is the working potential, ∆*V*_mean_ (V) is the mean of working potential, and ∆*t* (s) is the discharge time for electrode materials.

Prior to the assembly of the quasi‐solid‐state ASC device, a PVA–KOH gel electrolyte was prepared by dissolving 3 g of PVA in 1 m KOH (30 mL) of aqueous solution.^79^ The obtained mixture was heated to 90 °C under vigorous stirring until a clear gel was formed. The MoO_2_@NC and CuCo_2_S_4_ electrodes were coated with filter paper as a separator and PVA–KOH as a gel electrolyte and stacked to form the MoO_2_@NC//CuCo_2_S_4_ device after solidification. The area, thickness, and volume of the assembled device were about 6 cm^2^ (2 cm × 3 cm), 0.2 cm, and 1.2 cm^3^, respectively. The specific capacitance (*C*_d_, F g^−1^), energy density (*E*, W h kg^−1^), and power density (*P*, W kg^−1^) of the device were calculated based on the following equations

(6) $$C_{d}\;=\;\frac{I_{s}\;\cdot \;\Delta t}{M\;\cdot \;\Delta V_{s}}$$

(7) $$E\;=\;\frac{C_{d}\;\cdot \;\Delta V_{s}{}^{2}}{7.2}$$

and

(8) $$P\;=\;\frac{3600\;\cdot \;E}{\Delta t_{s}}$$

where *I*_s_ (A) is the discharge current, ∆*V*_s_ (V) is the voltage window, and *M* (g) is the total mass of the electrodes.

## Conflicts of interest

The authors declare no conflict of interest.

## Supporting information

Supplementary
