# Supplementary material for: High‐Performance Flexible Quasi‐Solid‐State Supercapacitors Realized by Molybdenum Dioxide@Nitrogen‐Doped Carbon and Copper Cobalt Sulfide Tubular Nanostructures
Source: Adv Sci (Weinh). 2018 Aug 11;5(10):1800733. doi: 10.1002/advs.201800733 (PMC6193180; doi:10.1002/advs.201800733)
Supplement: Supplementary file 1 — Supplementary [file ADVS-5-1800733-s001.pdf]

Copyright WILEY-VCH Verlag GmbH & Co. KGaA, 69469 Weinheim, Germany, 2018.

Supporting Information

for *Adv. Sci.*, DOI: 10.1002/advs.201800733

High-Performance Flexible Quasi-Solid-State Supercapacitors Realized by Molybdenum Dioxide@Nitrogen-Doped Carbon and Copper Cobalt Sulfide Tubular Nanostructures

Shude Liu, Ying Yin, Kwan San Hui, Kwun Nam Hui, Su Chan Lee, and Seong Chan Jun**

Copyright WILEY-VCH Verlag GmbH & Co. KGaA, 69469 Weinheim, Germany, 2016.

Supporting Information

Title High-performance flexible quasi-solid-state supercapacitors realized by molybdenum dioxide@nitrogen-doped carbon and copper cobalt sulfide tubular nanostructures

Shude Liu,[1] Ying Yin,[2] Kwan San Hui,[3] Kwun Nam Hui,[,4] Su Chan Lee,[1] and Seong Chan Jun*[,1]*

S. Liu, Y. Yin, Prof. K. S. Hui, Prof. K. N. Hui, S. Lee, Prof. S. C. Jun

[1]School of Mechanical Engineering, Yonsei University, Seoul 120-749, South Korea.

[2]Guangxi Key Laboratory of Information Materials, Guilin University of Electronic Technology, Guilin 541004, PR China.

[3]School of Mathematics, University of East Anglia, Norwich, NR4 7TJ, United Kingdom.

[4]Institute of Applied Physics and Materials Engineering, University of Macau, Avenida da Universidade, Taipa, Macau, China.

*Corresponding author's E-mail: scj@yonsei.ac.kr

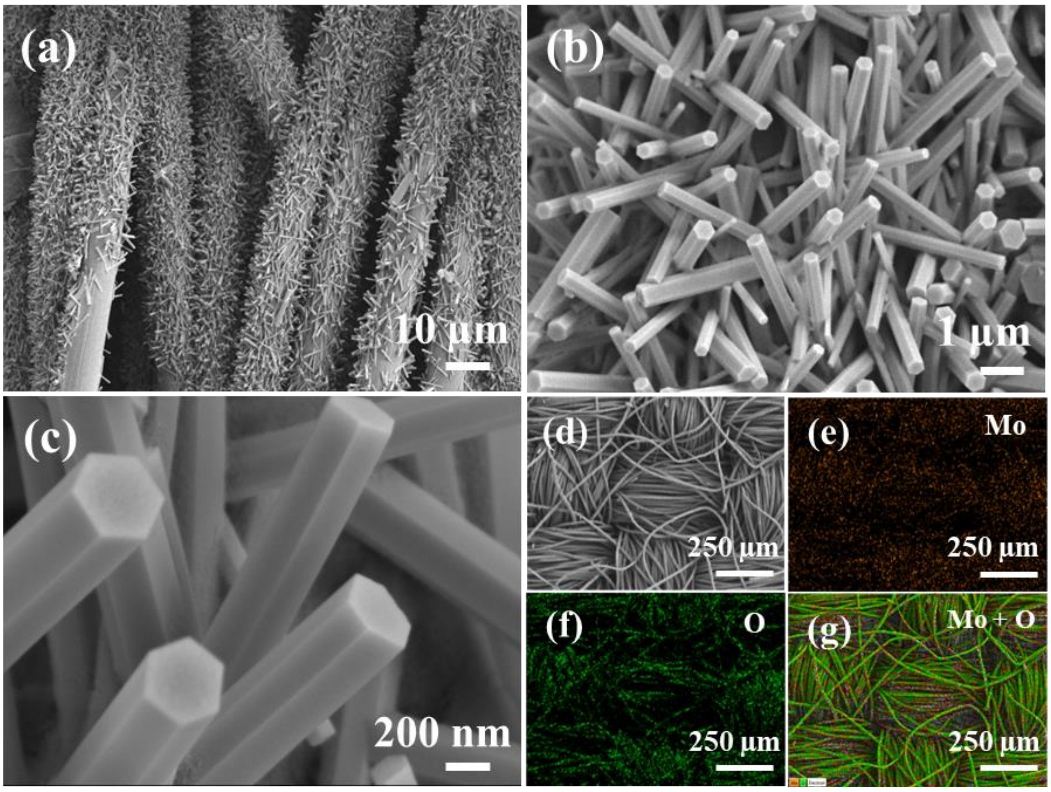

Figure S1. (a-d) FESEM images of MoO_3 nanorods at low and high magnifications. (e–g) EDS mapping images of Mo, O, and overlap elements in (d).

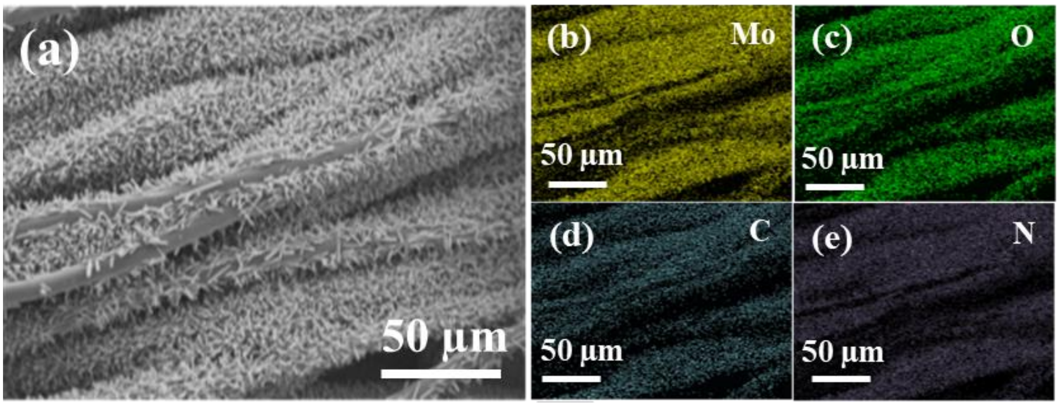

Figure S2. (a) FESEM image and (b-e) corresponding EDS mapping images of Mo, O, C, and N for MoO_2@NC nanorods.

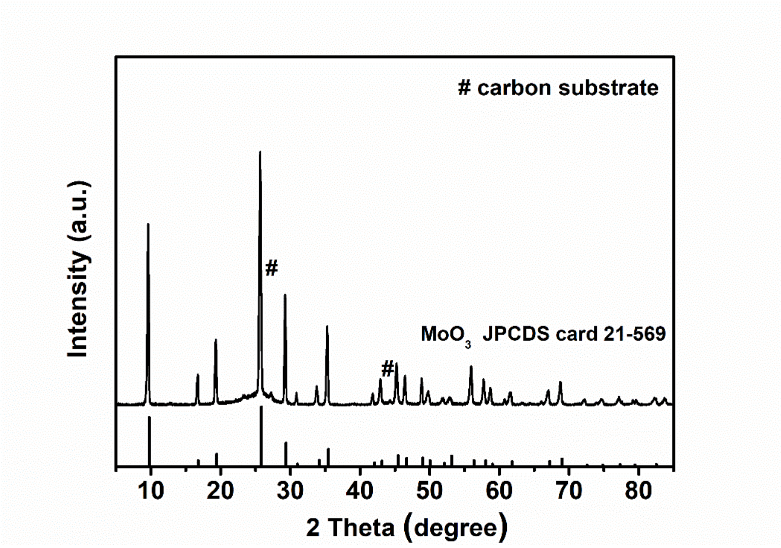

Figure S3.Typical XRD pattern of MoO_3 nanorods on carbon fiber.

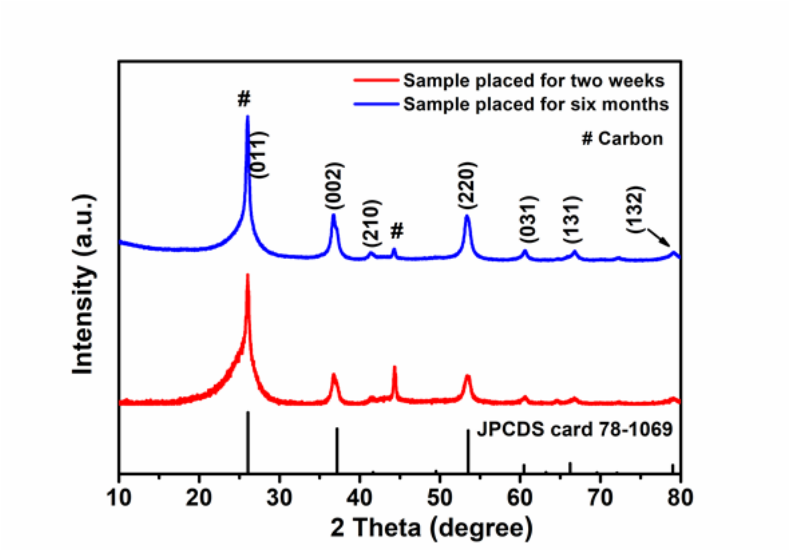

Figure S4. XRD patterns of as-prepared MoO_2@NC samples placed in air for two weeks and six months.

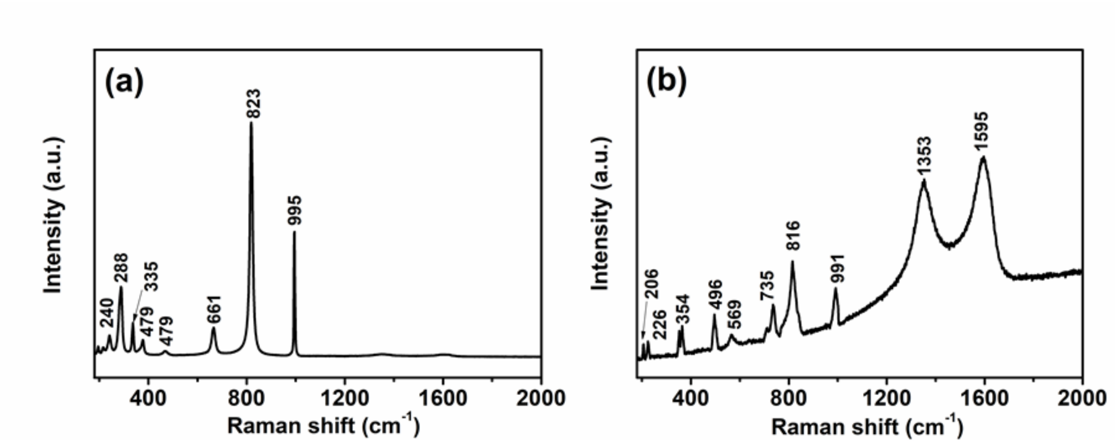

Figure S5. Raman spectra of MoO_3 nanorods and MoO_2@NC tubular nanostructure.

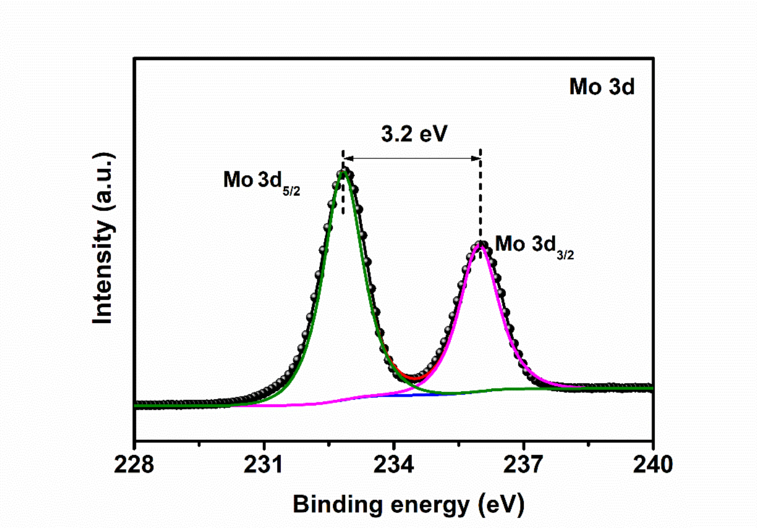

Figure S6. High-resolution Mo 3d spectrum of MoO_3 nanorods.

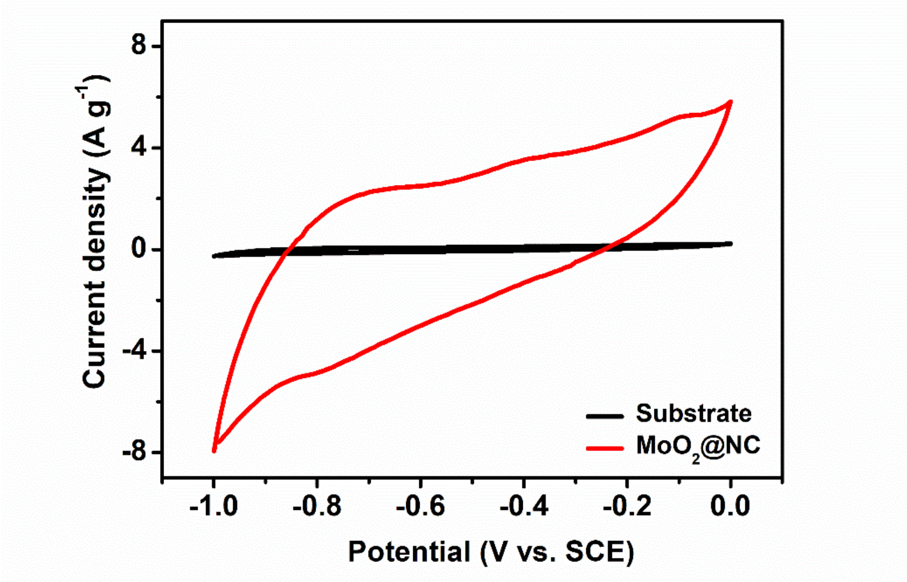

Figure S7. (a) Comparison of CV curves of pure carbon fiber and MoO_2@NC electrodes at a scan rate of 10 mV s^{-1}.

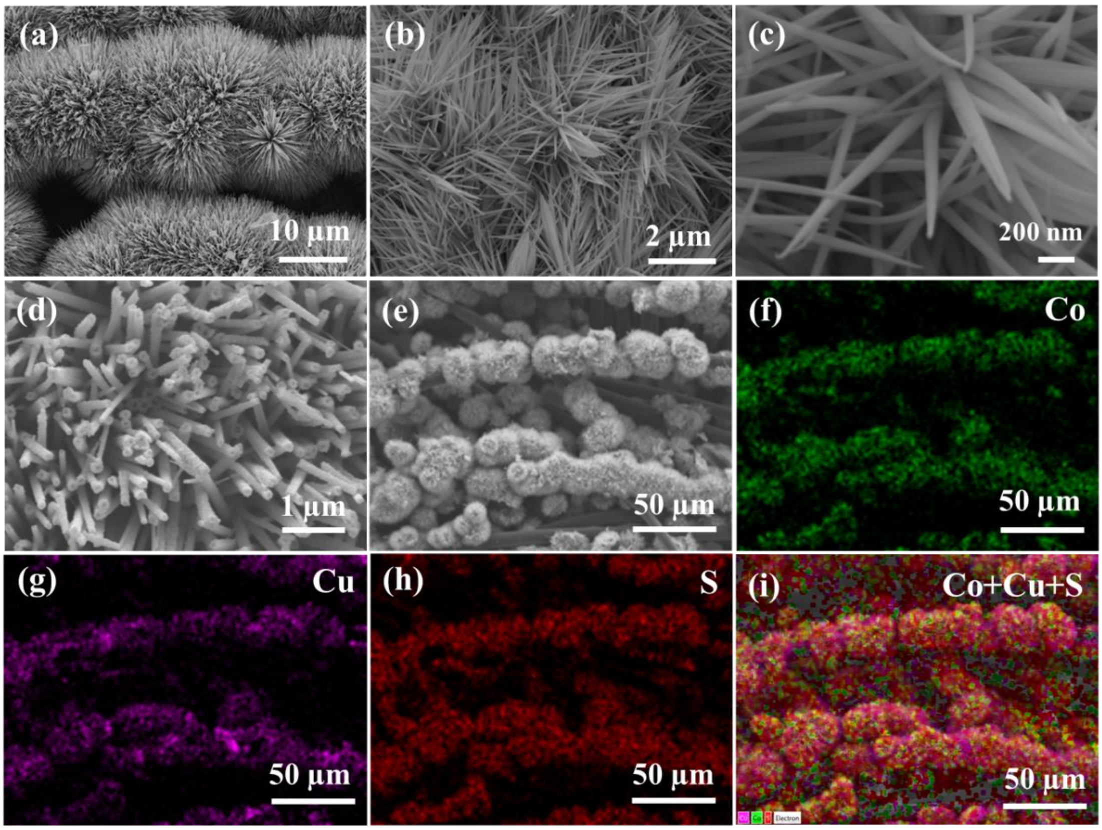

Figure S8. (a-c) FESEM images of Cu–Co precursor nanowires. (d, e) FESEM images and (f-h) the corresponding EDS mapping images of Co, Cu, and S and (i) overlap elements in image (e) for $CuCo_2S_4$.

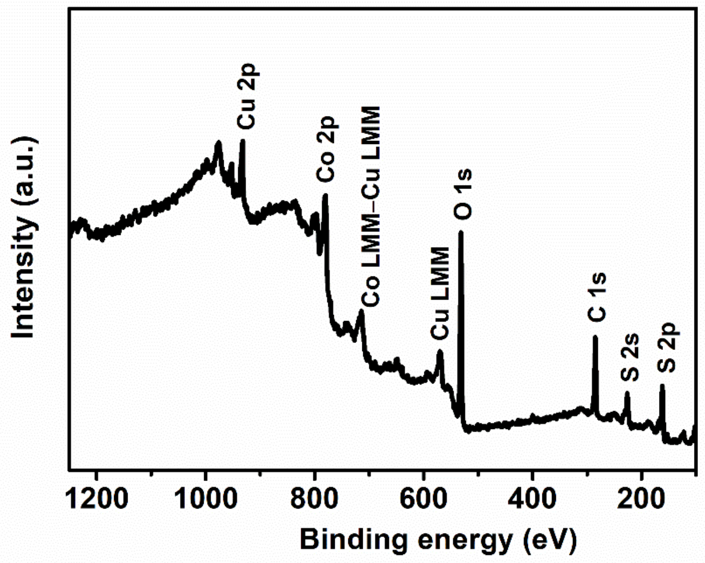

Figure S9. Full XPS spectrum of the $CuCo_2S_4$ nanostructure.

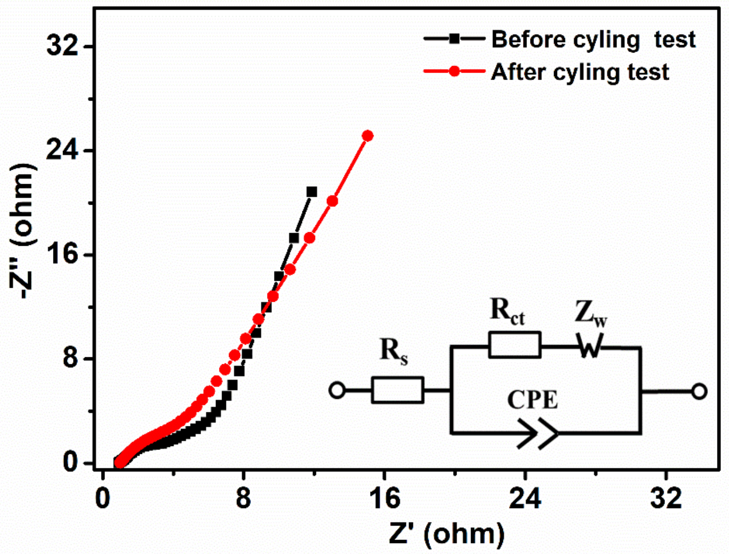

Figure S10. EIS curves of the $CuCo_2S_4$ nanostructure before and after cycling tests.

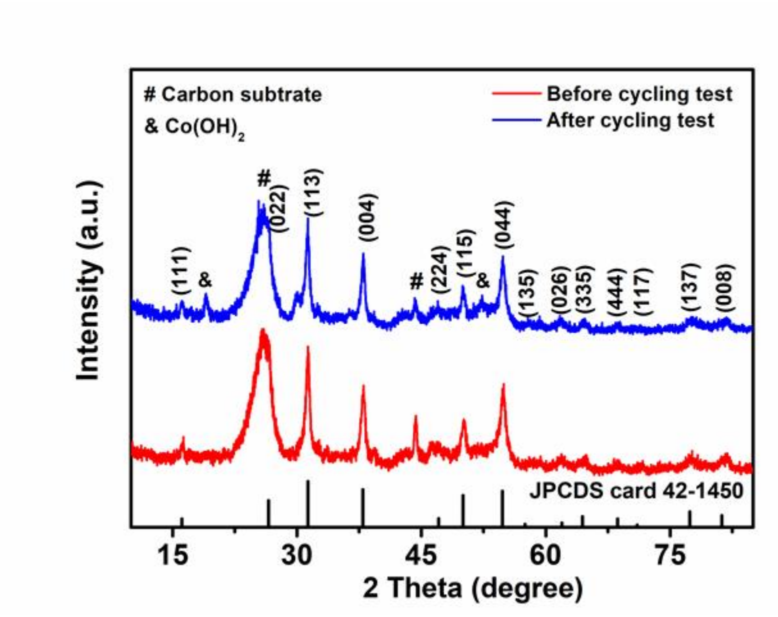

Figure 11. XRD patterns of as-prepared $CuCo_2S_4$ samples before and after cycling test.

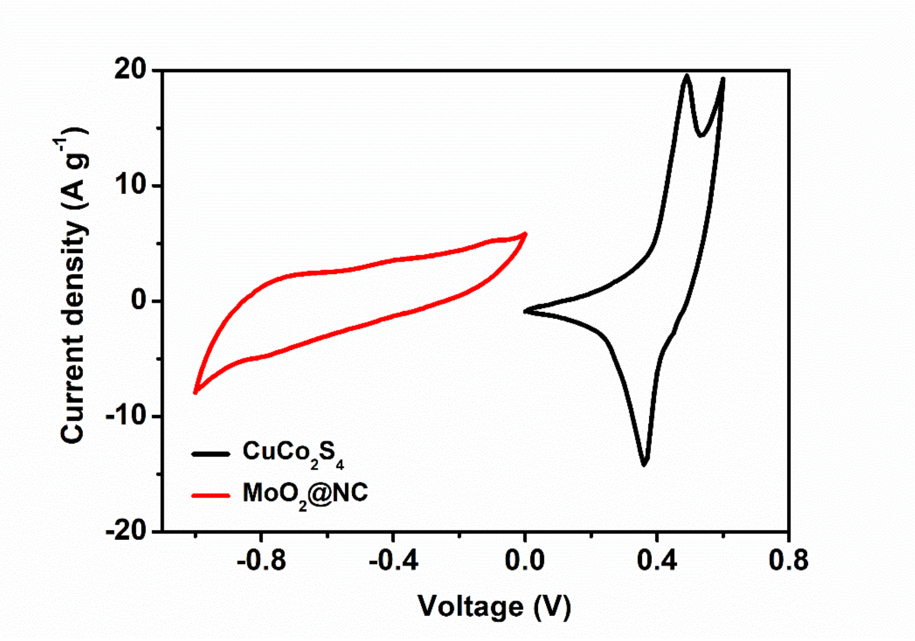

Figure S12. Comparative CV curves obtained for the MoO_2@NC and $CuCo_2S_4$ nanostructures in aqueous 1 M KOH at a scan rate of 10 mV s^{-1}.

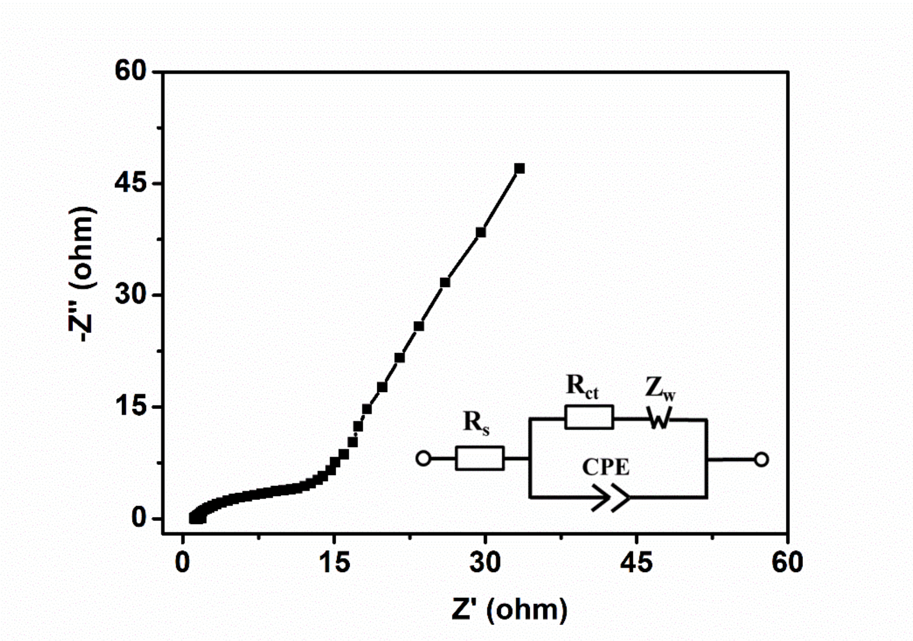

Figure S13. EIS curves of the MoO_2@NC//$CuCo_2S_4$ ASC device.

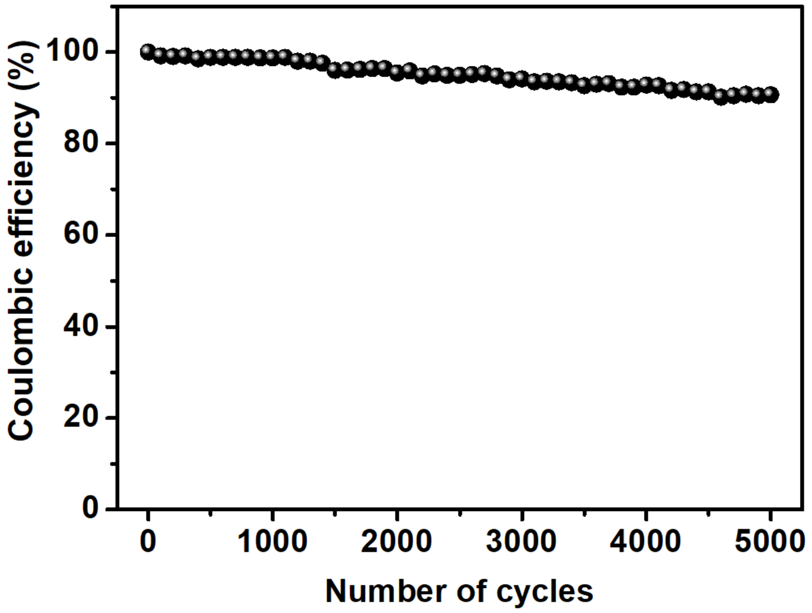

Figure S14. The Coulombic efficiency of the MoO_2@NC//$CuCo_2S_4$ ASC device during cycling test.

Table S1. Comparison of specific capacitance of reported MoO_2-based materials and the present MoO_2@NC tubular nanostructure.

Materials	Specific capacitance	Current density/scan rate	Electrolyte	Ref.
mesoporous MoO_2	146 F g^{-1}	5 mV s^{-1}	1 M LiOH	S[1]
carbon-encapsulated MoO_2 nanofilms	208.4 F g^{-1}	3 mA cm^{-2}	1 M LiOH	S[2]
helical MoO_2 particles	174.97 mF cm^{-2}	1.43 mA cm^{-2}	1 M Na_2SO_4	S[3]
MoO_2 particles	318 F g^{-1}	0.5 A g^{-1}	2 M KOH	S[4]
MoO_2 nanoparticles/CNTs	467.4 F g^{-1}	1 A g^{-1}	2 M KOH	S[5]
porous graphene/MoO_2 nanoparticles	356 F g^{-1}	0.1 A g^{-1}	1 M KOH	S[6]
graphene/MoO_2 nanoparticles	404 F g^{-1}	2 mV s^{-1}	1 M Na_2SO_4	S[7]
MoO_2@rGO nanocomposite	298.4 F g^{-1}	0.04 A g^{-1}	$NaPF_6$	S[8]
MoO_2@NC tubular nanostructure	548 F g^{-1}	1 A g^{-1}	1 M KOH	Present work

References

1. X. Li, J. Shao, J. Li, L. Zhang, Q. Qu and H. Zheng, *J. Power Sources*,, **2013**, *237*, 80-83.
2. Y. Li, F. Tang, R. Wang, C. Wang and J. Liu, *ACS Appl. Mater. Interfaces*, **2016**, *8*, 30232-30238.
3. X.-F. Lu, Z.-X. Huang, Y.-X. Tong and G.-R. Li, *Chem. Sci*, **2016**, *7*, 510-517.
4. H. Xuan, Y. Zhang, Y. Xu, H. Li, P. Han, D. Wang and Y. Du, *Phys. Status Solidi A*, **2016**, *213*, 2468-2473.
5. J.-Z. Wu, X.-Y. Li, Y.-R. Zhu, T.-F. Yi, J.-H. Zhang and Y. Xie, *Ceram Int.*, **2016**, *42*, 9250-9256.
6. L. Zhang, H. Lin, L. Zhai, M. Nie, J. Zhou and S. Zhuo, *J. Mater. Sci*, **2017**, *32*, 292-300.
7. X. Mu, X. Liu, K. Zhang, J. Li, J. Zhou, E. Xie and Z. Zhang, *Electron Mater Lett*, **2016**, *12*, 296-300.
8. K. Ramakrishnan, C. Nithya and R. Karvembu, *ACS Appl. Energy Interfaces*, **2018**, *1*, 841–850.
